# Association between bacterial homoplastic variants and radiological pathology in tuberculosis

**DOI:** 10.1136/thoraxjnl-2019-213281

**Published:** 2020-06-15

**Authors:** Louis Grandjean, Joha Monteserin, Robert Gilman, Julia Pauschardt, Sakib Rokadiya, Cesar Bonilla, Viviana Ritacco, Julia Rios Vidal, Julian Parkhill, Sharon Peacock, David AJ Moore, Francois Balloux

**Affiliations:** 1 Department of Medicine, Imperial College London, London, UK; 2 Laboratorio de Investigacion y Enfermedades Infecciosas, Cayetano Heredia Pervuvian University, Lima, Peru; 3 Institute of Child Health, UCL Division of Infection and Immunity, London, UK; 4 Instituto Nacional de Enfermedades Infecciosas INEI-ANLIS, Administración Nacional de Laboratorios e Institutos de Salud Dr Carlos G Malbrán, Buenos Aires, Argentina; 5 Johns Hopkins Bloomberg School of Public Health, Johns Hopkins University, Baltimore, Maryland, USA; 6 Faculty of Medicine, Imperial College London, London, UK; 7 Unidad Tecnica de Tuberculosis MDR, Ministerio de Salud, Lima, Peru; 8 Pathogen Genomics Group, Wellcome Trust Sanger Institute, Cambridge, UK; 9 Faculty of Medicine, University of Cambridge, Cambridge, UK; 10 TB Centre, London School of Hygiene and Tropical Medicine, London, UK; 11 UCL Genetics Institute, University College London, London, UK

**Keywords:** tuberculosis, imaging/CT MRI etc, clinical epidemiology

## Abstract

**Background:**

Understanding how pathogen genetic factors contribute to pathology in TB could enable tailored treatments to the most pathogenic and infectious strains. New strategies are needed to control drug-resistant TB, which requires longer and costlier treatment. We hypothesised that the severity of radiological pathology on the chest radiograph in TB disease was associated with variants arising independently, multiple times (homoplasies) in the *Mycobacterium tuberculosis* genome.

**Methods:**

We performed whole genome sequencing (Illumina HiSeq2000 platform) on *M. tuberculosis* isolates from 103 patients with drug-resistant TB in Lima between 2010 and 2013. Variables including age, sex, HIV status, previous TB disease and the percentage of lung involvement on the pretreatment chest radiograph were collected from health posts of the national TB programme. Genomic variants were identified using standard pipelines.

**Results:**

Two mutations were significantly associated with more widespread radiological pathology in a multivariable regression model controlling for confounding variables (Rv2828c.141, RR 1.3, 95% CI 1.21 to 1.39, p<0.01; rpoC.1040 95% CI 1.77 to 2.16, RR 1.9, p<0.01). The rpoB.450 mutation was associated with less extensive radiological pathology (RR 0.81, 95% CI 0.69 to 0.94, p=0.03), suggestive of a bacterial fitness cost for this mutation in vivo. Patients with a previous episode of TB disease and those between 10 and 30 years of age also had significantly increased radiological pathology.

**Conclusions:**

This study is the first to compare the *M. tuberculosis* genome to radiological pathology on the chest radiograph. We identified two variants significantly positively associated with more widespread radiological pathology and one with reduced pathology. Prospective studies are warranted to determine whether mutations associated with increased pathology also predict the spread of drug-resistant TB.

**Key messages**

**What is the key question?**

- Is there an association between bacterial genotype and pathology in drug-resistant TB?

**What is the bottom line?**

- This study demonstrates that polymorphisms in Rv2828c and rpoC are independently associated with more widespread pathology on the pretreatment chest radiograph of patients with drug-resistant TB.

**Why read on?**

- As the first study to compare the **Mycobacterium* tuberculosis* genome to radiological pathology on the chest radiograph, it improves our understanding of how pathogen genetic factors contribute to pathology in TB, which could enable tailored treatments to the most pathogenic and infectious strains.

## Introduction

Pathology in TB disease depends on host, pathogen and environmental factors. Understanding to what extent pathogen genetic factors contribute to the variability in pulmonary pathology due to *Mycobacterium tuberculosis* could allow those patients that harbour the most pathogenic and thereby infectious organisms^1 2^ to be identified for enhanced treatments, transmission prevention measures and contact tracing. Multidrug-resistant tuberculosis (MDRTB) treatment outcomes are worse, medications are more toxic and the cost of treatment is 10 times greater than drug-susceptible TB.^3^ This makes prevention of new cases of MDRTB particularly important. Although there is now a greater understanding of which polymorphisms are associated with drug resistance in TB, relatively little is known about the consequences of the genetic makeup of the pathogen on disease severity and progression in vivo. So far, no studies have examined the association of the *M. tuberculosis* pathogen genome with radiologically quantified pathology on the chest radiograph.

Many studies have implicated the influence of *M. tuberculosis* lineage with TB pathogenesis in laboratory animals; however, these studies are limited by comparison to laboratory strains and typically lack information about whole genome variability.^4–6^ It is well established that there is a fitness cost arising from some rifampicin resistance conferring mutations in some drug-resistant strains in vitro,^7^ and drug-resistant strains have been shown to cause less pathology in guinea pigs^8^ than controls. However, Comas **et al** demonstrated that this fitness cost in *M. tuberculosis* when measured in vitro by competitive fitness assays can be mitigated by compensatory mutations in the rpoA and rpoC genes.^9^ The heterogeneity in the fitness cost of drug resistance mutations is also key to the future predictions of MDRTB spread.^10 11^

Convergent evolutionary (homoplastic) mutations emerging recurrently and independently at the same locus in multiple clades of the phylogeny form a subset of mutations likely to have been affected by strong directional natural selection. These homoplastic mutations represent excellent candidate mutations for polymorphisms involved in antimicrobial resistance, immune evasion and virulence^12 13^ and may influence radiological pathology and outcome.

Work by Ralph and colleagues^14^ has demonstrated that assessment of the degree of pulmonary parenchymal involvement on the chest radiograph can be reliably reproduced by independent observers with a high agreement between observers and that this is predictive of the sputum smear result at 2 months. We hypothesised that some convergent evolutionary polymorphisms could be significantly associated with lung pathology as quantified by the extent of pulmonary parenchymal involvement on the pretreatment diagnostic chest radiograph. In order to test this hypothesis, we performed whole genome sequencing on 103 TB patients with drug-resistant disease in Lima who had a chest radiograph performed before the start of treatment.

## Materials and methods

### Field methods, culture techniques and sample selection

Collection of clinical and demographic variables, sampling of sputa, culture and DNA extraction were undertaken as previously described.^15^ Briefly, samples with available pretreatment chest radiographs were selected from a prospective cohort study^16^ undertaken in the regions of Callao and Lima South between 2010 and 2013. Demographic data such as age and sex were collected from the clinical record at the health post. Previous TB disease was defined as any previous episode of clinically diagnosed and treated TB disease prior to the present episode. A pretreatment chest radiograph was defined as a radiograph taken before the onset of treatment for the current episode of TB disease at the same time as the initial diagnostic sampling. Testing for HIV was undertaken for all patients, and this result was confirmed from the clinical record. A bad treatment outcome was defined as those that died during treatment or had a bacteriological failure of treatment, while a good treatment outcome was defined as treatment completion or success as per the WHO guidelines.^17^ Those that transferred treatment outside the study area or those that abandoned treatment were regarded as unknown outcomes.

Sputum samples from all patients were transported to the regional reference laboratories and processed both on liquid (MODS) and solid Ogawa media. An aliquot of each positive culture was subcultured at Universidad Peruana Cayetano Heredia and DNA was extracted.^15^ All samples were retested and had drug susceptibility profiles confirmed at the national reference laboratory using the proportions method on agar.

### Reading of chest radiographs and quantification of pathology

Chest radiographs included in the analysis were all correctly labelled, posteroanterior, baseline pretreatment films. Poor quality radiographs were excluded if they were over or under penetrated, over rotated or with inadequate field of view. Chest radiographs were read independently and prior to data analysis as described^14 18^ by two experienced clinicians blinded to the sequencing data and specialised in managing TB. The total percentage of the lung opacified with pathology was used as the pathology score outcome as previous studies have demonstrated that this is the only reliably reproducible indicator between observers.^14^ Briefly, the lungs were divided into six equally sized zones and the percentage of opacity in each zone and overall was estimated to the nearest 5%.^18^ Readings from the two observers were compared to quantify the degree of correlation between observers and an average of the two readings was used for the analysis. In addition to Spearman’s rank correlation, a Bland-Altman plot was used to compare the agreement between reviewers.

### Genome sequence quality control

We prepared Illumina sequencing libraries with a 450 bp insert size, using the manufacturer’s protocols, and then undertook sequencing on an Illumina HiSeq2000 with paired-end reads of length of 100 bp. We multiplexed 96 samples per lane to attain an average depth of coverage of ~97-fold. We confirmed the species in the short reads using Kraken^19^ and then assembled the paired-end sequence reads with an improved assembly pipeline,^20^ based on Velvet.^21^ A list of isolates and their accession numbers in the European Nucleotide Archive is provided in online supplementary file 1 (project number: ERP004677). Following this, short reads were mapped to the corrected H37Rv reference genome available from Casali *et al* 22 genome.cshlp.org/content/suppl/2012/02/01/gr.128678.111.DC1/1_H37RvQM_embl.txt. In doing so, we employed SMALT V.0.7.4 (www.sanger.ac.uk/science/tools/smalt-0) using maximum and minimum insert sizes of 1000 and 50, respectively. To annotate single nucleotide polymorphisms (SNPs), we used SAMtools mpileup^23^ and BCFtools, as described by Harris *et al*.^24^ We included SNPs that were covered by at least two forward and two reverse short paired-end reads.^25^ A minimum base call quality of 50 and a minimum root mean squared mapping quality of 30 to call a SNP were used. Furthermore, the SNPs at sites with heterogeneous mapping where less than 75% of reads at that site covered the SNP were excluded from the analysis.^24^ We obtained the multiple alignment by generating pseudosequences, after ignoring small indels. Any site with >5% non-calls was excluded from the analysis, and an arbitrary minimum SNP prevalence threshold of 3% (3/103) and a homoplasy count of a minimum of three independent occurrences across the phylogenetic tree were chosen to avoid spurious comparison of low prevalence mutant strains.

10.1136/thoraxjnl-2019-213281.supp3 Supplementary data

### Phylogenetic analysis

A maximum likelihood tree was constructed with concatenated SNPs from the whole genome sequence data using R software (R Foundation for Statistical Computing, Vienna, Austria 2011, www.R-project.org) with the PHANGORN package.^26^ Clades were named according to the latest SNP-based bar code.^27^

### Identification of convergent evolutionary variants

Variants were identified using phyC software^12^ to determine the number of independent occasions in which an ancestral base was different from the descendent base at any given site in the tree.

### Sample size and model construction

As no previous study had been undertaken to provide an estimate of the expected effect size, formal power calculation was not performed. The study aim to recruit more than 100 patients was therefore based on pragmatic and systematic considerations. The extent of radiological pathology was Poisson distributed; therefore, a Poisson model was constructed using this variable as the dependent variable. Univariate analysis was first undertaken for each polymorphism. Polymorphisms with a p value of <0.05 on univariate analysis were selected for inclusion in a multivariable model together with confounding variables. The set of confounding variables that were included in the multivariable model was defined a priori. These included sex, age, previous TB disease, drug resistance status (mono-resistant or multidrug-resistant) and lineage. Multivariable Poisson regression was used to calculate the p values having corrected for confounding variables. The R package ‘stats’ was then used to plot the predicted pathology having accounted for other confounding variables in the multivariable model. All p values were Bonferroni corrected. Missing data were omitted and managed using the default ‘na.omit’ setting under the glm function in R. Following reviewers’ comments, we performed additional population level correction using principal components^28^ to account for the effect of lineage on the association between the homoplastic polymorphisms and radiological pathology. We also performed a subanalysis of our principal findings with and without the inclusion of patients with previous TB disease as a variable.

## Results

**Figure 1:**
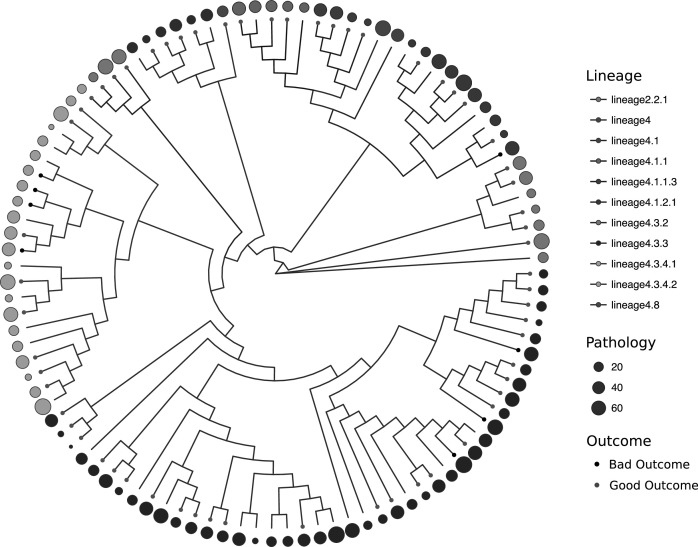
Study phylogeny with branches coloured by sublineage as defined by Coll *et al*. Clinical outcome following treatment is displayed (bad outcome: black dots, good outcome: grey dots on the tips of the tree) in a ring surrounding the phylogeny with the pathology on chest radiograph (% of lung with disease) represented by a circle of varying size according to the extent of pathology.

**Figure 2:**
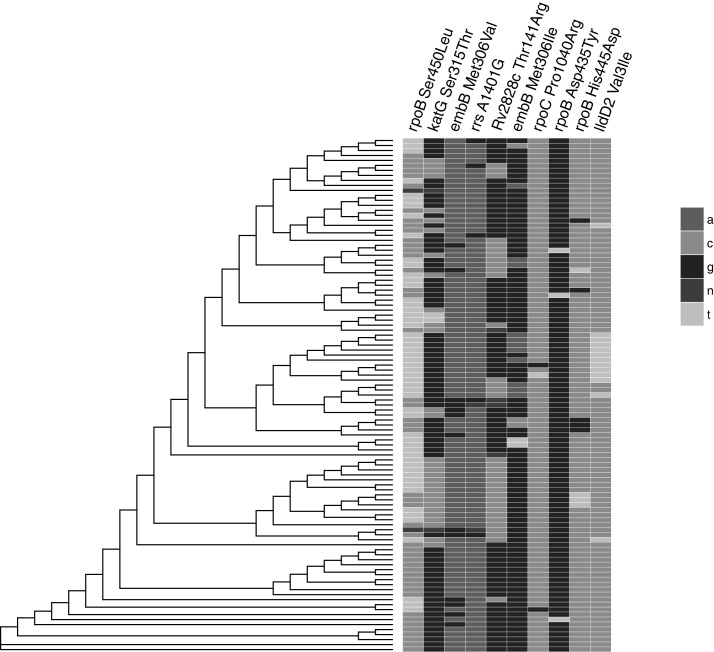
The distribution of homoplastic polymorphisms identified in the study. A, adenine, G, guanine, C, cytosine, T, thymine, N, unknown (no call).

A total of 103 different patients had radiological, clinical, demographic and pathogen data available for analysis (table 1). The proportion of patients with MDRTB was 88% (91/103) and 5% (5/103) were HIV positive. The predominant sublineage was lineage 4.3.3 (41%, 43/103) of the Euro-American lineage (Lineage 4). As a likely consequence of Chinese immigration to Peru in the mid-19th century to work on the railways and guano farms, 7% (7/103) of strains were from the Beijing family (table 1, figure 1, online supplementary figure 2). Evaluation of the extent of radiological pathology by two independent observers was significantly positively correlated using the Spearman rank correlation coefficient between observer pathology scores (Spearman ρ=0.66, 95% CI 0.53 to 0.76, p<0.001) (online supplementary figure 1). The mean difference in pathology scores between observers in the Bland-Altman analysis was 4.7%, while the upper limit of agreement was 43.1% (95% CI 36.7 to 43.1) and the lower limit −32.9% (95% CI −26.5 to −39.4). Pathology appeared evenly distributed by sublineage before considering confounding variables and there were eight bad outcomes (failed treatment or died during treatment) (8/103) according to the WHO definitions (figure 1). The distribution of homoplastic variants across the phylogenetic tree is shown in figure 2; the most frequently observed homoplastic mutation was the rpoB Ser450Leu mutation, which occurred independently 13 times with a prevalence of 54% in the study population (figure 2, table 2).

10.1136/thoraxjnl-2019-213281.supp1 Supplementary data

10.1136/thoraxjnl-2019-213281.supp2 Supplementary data

**Table 1:** Clinical, demographic and pathogen data for the 103 isolates included in the study

Variable	No. (%*†)
No. of patients	103
Male gender (%)	71 (69)
Age	
10–19	21 (20)
20–29	46 (45)
30–39	13 (13)
40–49	11 (11)
50–59	7 (7)
60–69	2 (2)
70–79	2 (2)
Missing	1 (1)
HIV positive	5 (5)
Radiological pathology % of lung involved (median/IQR)	30 (15–50)
Drug susceptibility status	
Mono-resistant	12 (12)
MDR	91 (88)
Previous tuberculosis disease	30 (29)
Lineage	
Lineage 2.2.1	7 (7)
Lineage 4	7 (7)
Lineage 4.1	1 (1)
Lineage 4.1.1	5 (5)
Lineage 4.1.1.3	4 (4)
Lineage 4.1.2.1	10 (10)
Lineage 4.3.2	4 (4)
Lineage 4.3.3	43 (42)
Lineage 4.3.4.1	4 (4)
Lineage 4.3.4.2	17 (16)
Lineage 4.8	1 (1)

*% total 101 due to rounding.

†No missing data unless stated.

MDR, multidrug resistant .

**Table 2:** Homoplastic mutations identified in the dataset

Reference position	Gene	Mutation	Homoplasies (n)	No. (Prevalence)	Reference base	Mutant base
761160	*rpoB*	Ser450Leu	13	54 (52%)	C	T
2155176	*katG*	Ser315Thr Ser315Asn	10	71 (69%)	C	G/T
4247436	*embB*	Met306Val	9	13 (13%)	A	G
1473254	*rrs*	A1401G rRNA gene	5	5 (5%)	A	G
3135920	*Rv2828c*	Thr141Arg	4	34 (33%)	G	C
4247438	*embB*	Met306Ile	4	16 (16%)	G	A/C/T
766493	*rpoC*	Pro1040Arg Pro1040Leu	3	3 (3%)	C	G/T
761114	*rpoB*	Asp435Tyr	3	3 (3%)	G	T
761144	*rpoB*	His445Asp His445Tyr	3	9 (9%)	C	G/T
2123153	*lldD2*	Val3Ile	3	13 (13%)	C	T

### Clinical and demographic variables associated with chest radiograph pathology

**Figure 3:**
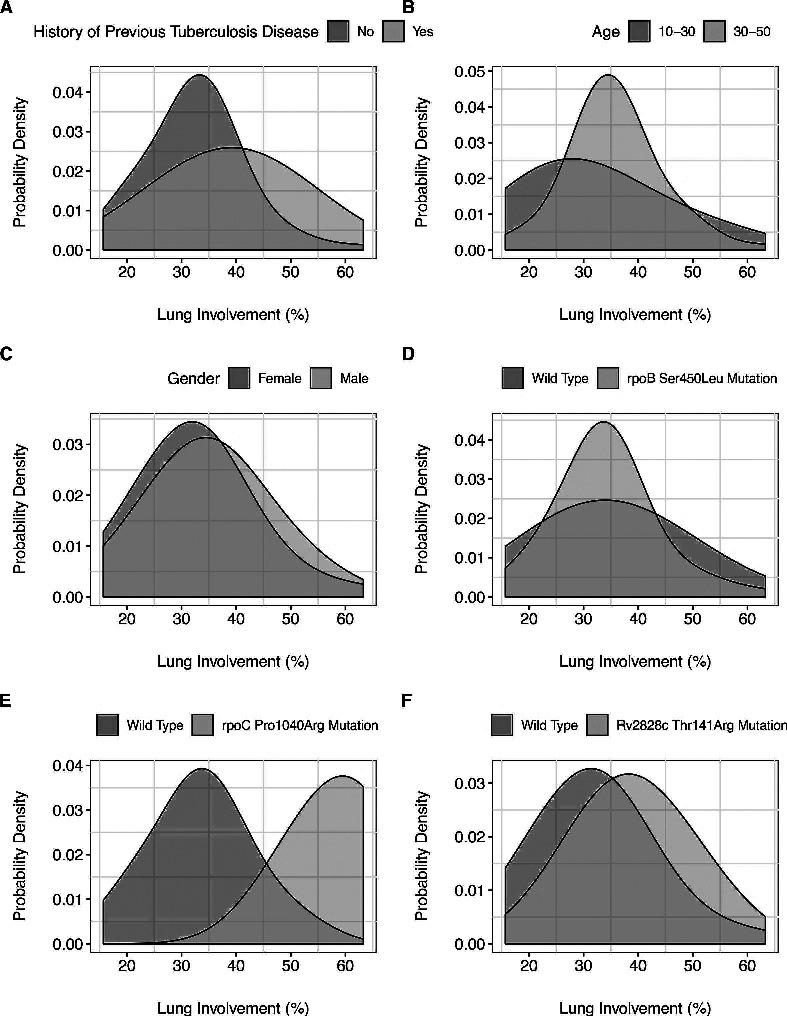
Distribution of radiograph pathology as a function of epidemiological and pathogen genetic variables; (A) previous episode of TB, rate ratio 1.2, p=<0.01; (B) age range 10–30 versus other ages, rate ratio 1.2, p<0.01; (C) gender, rate ratio 1.13, p=0.29; (D) rpoB 450; Rr 0.8, p=0.03; (E) rpoC.1040, Rr 1.97, p<0.01; (F) Rv2828c.141, Rr 1.3, p<0.01.

A previous episode of TB disease was associated with more extensive pathology on the chest radiograph (RR 1.2, p<0.01, 95% CI 1.10 to 1.28; figure 3A). Those aged between 30 and 39 had less severe disease than those between 10 and 19 years of age (RR 0.66, p<0.01, 95% CI 0.50 to 0.82). When age as a variable in the multivariable model was trichotomised into those less than 30, between 30 and 50 years old and those greater than 50 years of age, those less than 30 years of age were shown to have more severe disease than those between 30 and 50 years of age (RR 1.3, p<0.01, 95% CI 1.17 to 1.38, figure 3B). There was no significant difference in pathology observed by sex (figure 3C). HIV infection was not statistically associated with more severe disease.

### Association between homoplastic polymorphisms and chest radiograph pathology

We detected 10 homoplastic polymorphisms in 103 strains. Other than the *rrs* A1401G mutation that occurred in an rRNA gene, all homoplastic polymorphisms detected in the dataset were non-synonymous (amino acid changing). Seven homoplastic polymorphisms were associated with the extent of lung pathology with a p value<0.05 on univariate analysis and these were included in the multivariable regression together with clinical and sociodemographic confounding variables. Three mutations were significantly associated with radiological pathology after controlling for confounding variables and Bonferroni correction (table 3).

**Table 3:** Variables in the multivariable predictive regression model of radiological pathology in multidrug-resistant tuberculosis with rate ratios and their associated lower and upper 95% CI, SE and p values

Variable	Rate ratio	Upper CI	Lower CI	SE	P value*
Mutation					
katG.315	1.14	1.28	1.01	0.07	1.00
rpoB.450	0.81	0.94	0.69	0.06	0.03
rpoC.1040	1.97	2.16	1.77	0.10	<0.01
rpoB.435	1.21	1.40	1.01	0.10	1.00
Rv2828c.141	1.30	1.39	1.21	0.04	<0.01
embB.306	0.86	0.97	0.74	0.06	0.27
rpoB.445	1.09	1.25	0.93	0.08	1.00
Male gender	1.13	1.23	1.04	0.05	0.29
HIV positive	0.84	1.02	0.66	0.09	1.00
Age 10–19	Comparison	Comparison	Comparison	Comparison	Comparison
Age 20–29	0.97	1.08	0.87	0.05	1.00
Age 30–39	0.66	0.82	0.50	0.08	<0.01
Age 40–49	0.93	1.08	0.78	0.08	1.00
Age 50–59	1.20	1.37	1.02	0.09	1.00
Age 60–69	0.71	1.04	0.38	0.17	1.00
Age 70–79	0.43	0.79	0.07	0.18	<0.01
MDR	Comparison	Comparison	Comparison	Comparison	Comparison
Mono-resistant	0.86	0.99	0.72	0.07	0.75
Previous TB	1.19	1.28	1.10	0.05	<0.01
Lineage 2	Comparison	Comparison	Comparison	Comparison	Comparison
Lineage 4	1.28	1.49	1.08	0.10	0.48
Lineage 4.1.1	1.32	1.54	1.10	0.11	0.33
Lineage 4.1.1.3	1.06	1.34	0.78	0.14	1.00
Lineage 4.1.2.1	1.34	1.52	1.16	0.09	0.05
Lineage 4.3.2	1.15	1.40	0.89	0.13	1.00
Lineage 4.3.3	1.02	1.18	0.87	0.08	1.00
Lineage 4.3.4.1	0.61	0.89	0.33	0.14	0.02
Lineage 4.3.4.2	1.26	1.43	1.08	0.09	0.27
Lineage 4.8	0.98	1.39	0.57	0.21	1.00

*Bonferroni-corrected p value.

The Pro1040Lys mutation in rpoC had the greatest effect size on radiological pathology with a rate ratio of 1.9 (95% CI 1.77 to 2.16, p<0.01; figure 3E). Patients that harboured a strain with this mutation had a mean pathology score almost twice that of those without the mutation. This was independent of other confounding variables, although, the prevalence of the rpoC.1040 mutation was only at a frequency of 3%, limiting its influence at the population level. In contrast, the Rv2828c Thr141Arg mutation occurred in 34% of the population at a rate ratio of 1.3 (95% CI 1.21 to 1.39, figure 3F), meaning that those with disease caused by a strain with the mutation had 30% more likely to have more widespread pathology than those without. The most prevalent homoplastic mutation rpoB S450L (known to be associated with rifampicin resistance) was associated with decreased pathology (rate ratio 0.8, p=0.03, 95% CI 0.69 to 0.94; figure 3D). However, the protective association of this rpoB.450 polymorphism on radiological pathology was not statistically significant when additional correction for population structure with principal component analysis (PCA) was performed, demonstrating that the association may be confounded by lineage. Neither did this polymorphism remains statistically significantly protective of pathology when a subanalysis was performed on only TB naïve patients, suggesting that this association was influenced by prior disease status.

## Discussion

This is the first published study to compare pathogen genome-wide data with radiological pathology in *M. tuberculosis*. We identified that MDRTB patients with disease caused by a pathogen harbouring the Rv2828c.141 mutation had 30% increased risk of more extensive radiological pathology. Those MDRTB patients with disease caused by strains carrying the rpoC.1040 polymorphism were twice as likely to have more extensive radiological disease. Conversely, the rpoB.450 mutation was 20% protective of radiological pathology, although this association was not observed to be independent of lineage nor to prior TB after additional PCA correction and subanalysis.

The finding that strains with the rifampicin resistance rpoB.S450L mutation were associated with less widespread pathology and those with the rpoC.1040 polymorphism with more widespread pathology supports the notion that rifampicin resistance mediated by the rpoB.450 mutation is associated with a fitness cost that is compensated for by secondary mutations in rpoC.^9^ Interestingly, no evidence of a fitness cost as manifest by radiological pathology score was found for the katG.315 mutation that confers isoniazid resistance in *M. tuberculosis*, which may explain why this mutation is often found in multicase households.^29^

The third mutation significantly associated with lung pathology (Rv2828c.141) falls in the *Rv2828* gene, which is part of a toxin–antitoxin system together with vapBC22. Patients infected with strains carrying the homoplastic Rv2828c.141 mutation had on average 30% more severe pathology. The *vapB22*, *vapC22* and the *Rv2828* genes were all significantly upregulated in drug-resistant South African Beijing strains relative to drug susceptible control strains, suggesting that the action of these genes is indeed important in the survival of drug-resistant TB strains.^30^ An identical mutation in *Rv2828* at codon position 141 was also selected when the cell wall of susceptible TB strains was stressed by a novel antituberculous coumarin compound.^31^ Farhat *et al,* in a global strain collection, identified this gene to be under significant positive selection, suggesting that the gene is a site of adaptive advantageous evolution.^12^

It is self-evident that previous TB disease will be associated with more widespread pathology and our data also support this. It is not clear whether patients coinfected with TB-HIV more readily transmit TB or have increased disease severity.^32^ The number of HIV-infected patients in our study was low and the difference in disease severity was not found to be statistically significant.

The extent of radiological pathology on chest radiograph is predictive of smear positivity at 2 months into treatment^14^; Wells and Riley in their seminal studies of TB transmission describe bacterial burden as a predictor of infectiousness, while clinicians use the extent of parenchymal involvement as well as bacterial burden to predict the likely infectiousness of the patient. Understanding which polymorphisms are associated with extensive pathology may therefore aid clinicians in determining index case infectiousness and the likelihood of developing a secondary case of disease. Whether these polymorphisms are associated with the transmission of MDRTB requires prospective cohort studies of contacts exposed to wild type and mutant disease.

This study was strengthened by the use of bacterial whole genome sequencing, the ability to make comparison to pretreatment chest radiographs and the availability of potentially confounding variables to adjust the findings in the context of a multivariable model. However, it is clear that the causes of radiological pathology at diagnosis are multifactorial. Although ‘the delay to diagnosis’ is an unreliable variable to capture with often vague symptom start dates, this study is limited by the lack of data for this important confounding variable. Similarly, the socioeconomic status of the patient is likely to influence both the delay to presentation and adherence to prior treatment,^33^ making this a variable also likely to be associated with pathology on the chest radiograph. For those patients with previous TB disease, we did not capture data on the phenotype of the previous disease. However, a subanalysis restricted solely to TB naïve patients did not alter the principal findings.

The Bland-Altman comparison between observers demonstrated a 4.7% mean difference in pathology scores and the Spearman rank correlation between observers was significant. However, there was an increased difference between observers at the higher pathology scores. Future studies could improve the interobserver agreement by calibrating both observers on a training data set or employing AI pattern recognition algorithms instead from the outset.

In summary, this study provides a novel and interesting insight into the association of bacterial homoplastic polymorphisms with the extent of pathology on the chest radiograph following infection with *M. tuberculosis*. With improved statistical power, it may be possible to identify a larger set of bacterial genetic markers that help predict pathology, disease outcome and transmission. Prospective cohort studies are also warranted to determine if polymorphisms in the bacterial genome of the index case such as these are independently predictive of secondary cases of disease and the development of severe radiological pathology among close contacts.

## References

[1] Wells WF, Ratcliffe HL, Grumb C On the mechanics of droplet nuclei infection; quantitative experimental air-borne tuberculosis in rabbits. Am J Hyg 1948;47:11–28. 10.1093/oxfordjournals.aje.a119179 18921435

[2] Riley RL, Wells WF, Mills CC, et al Air hygiene in tuberculosis: quantitative studies of infectivity and control in a pilot ward. Am Rev Tuberc 1957;75:420–31. 10.1164/artpd.1957.75.3.420 13403171

[3] Suárez PG, Floyd K, Portocarrero J, et al Feasibility and cost-effectiveness of standardised second-line drug treatment for chronic tuberculosis patients: a national cohort study in Peru. Lancet 2002;359:1980–9. 10.1016/S0140-6736(02)08830-X 12076553

[4] Tsenova L, Ellison E, Harbacheuski R, et al Virulence of selected Mycobacterium tuberculosis clinical isolates in the rabbit model of meningitis is dependent on phenolic glycolipid produced by the bacilli. J Infect Dis 2005;192:98–106. 10.1086/430614 15942899

[5] Dormans J, Burger M, Aguilar D, et al Correlation of virulence, lung pathology, bacterial load and delayed type hypersensitivity responses after infection with different Mycobacterium tuberculosis genotypes in a BALB/c mouse model. Clin Exp Immunol 2004;137:460–8. 10.1111/j.1365-2249.2004.02551.x 15320894 PMC1809137

[6] Parwati I, van Crevel R, van Soolingen D Possible underlying mechanisms for successful emergence of the Mycobacterium tuberculosis Beijing genotype strains. Lancet Infect Dis 2010;10:103–11. 10.1016/S1473-3099(09)70330-5 20113979

[7] Andersson DI, Hughes D Antibiotic resistance and its cost: is it possible to reverse resistance? Nat Rev Microbiol 2010;8:260–71. 10.1038/nrmicro2319 20208551

[8] Barnett M, Busby SR, Mitchison DA Tubercle bacilli resistant to isoniazid: virulence and response to treatment with isoniazid in guinea-pigs and mice. Br J Exp Pathol 1953;34:568–81. 13106225 PMC2073524

[9] Comas I, Borrell S, Roetzer A, et al Whole-genome sequencing of rifampicin-resistant Mycobacterium tuberculosis strains identifies compensatory mutations in RNA polymerase genes. Nat Genet 2012;44:106–10. 10.1038/ng.1038 PMC3246538 22179134

[10] Durão P, Balbontín R, Gordo I Evolutionary mechanisms shaping the maintenance of antibiotic resistance. Trends Microbiol 2018;26:677–91. 10.1016/j.tim.2018.01.005 29439838

[11] Cohen T, Murray M Modeling epidemics of multidrug-resistant M. tuberculosis of heterogeneous fitness. Nat Med 2004;10:1117–21. 10.1038/nm1110 15378056 PMC2652755

[12] Farhat MR, Shapiro BJ, Kieser KJ, et al Genomic analysis identifies targets of convergent positive selection in drug-resistant Mycobacterium tuberculosis. Nat Genet 2013;45:1183–9. 10.1038/ng.2747 23995135 PMC3887553

[13] Grandjean L, Gilman RH, Iwamoto T, et al Convergent evolution and topologically disruptive polymorphisms among multidrug-resistant tuberculosis in Peru. PLoS One 2017;12:e0189838. 10.1371/journal.pone.0189838 29281674 PMC5744980

[14] Ralph AP, Ardian M, Wiguna A, et al A simple, valid, numerical score for grading chest X-ray severity in adult smear-positive pulmonary tuberculosis. Thorax 2010;65:863–9. 10.1136/thx.2010.136242 20861290

[15] Ausubel FM, Struhl K, Smith JA, et al A compendium of methods from current protocols in molecular biology : Ausubel FM, Brent E-R, Short protocols in molecular biology. John Wiley & Sons Inc, 1992 ISBN: 9780471577355 https://www.abebooks.co.uk/Short-Protocols-Molecular-Biology-Compendium-Methods/9011140108/bd

[16] Grandjean L, Gilman RH, Martin L, et al Transmission of multidrug-resistant and drug-susceptible tuberculosis within households: a prospective cohort study. PLoS Med 2015;12:e1001843. 10.1371/journal.pmed.1001843 26103620 PMC4477882

[17] World Health Organization, Stop TB Initiative (World Health Organization), Treatment of tuberculosis: guidelines. 4th edn Geneva: World Health Organization, 2010.

[18] Kriel M, Lotz JW, Kidd M, et al Evaluation of a radiological severity score to predict treatment outcome in adults with pulmonary tuberculosis. Int J Tuberc Lung Dis 2015;19:1354–60. 10.5588/ijtld.15.0098 26467588

[19] Wood DE, Salzberg SL Kraken: ultrafast metagenomic sequence classification using exact alignments. Genome Biol 2014;15:R46. 10.1186/gb-2014-15-3-r46 24580807 PMC4053813

[20] Page AJ, De Silva N, Hunt M, et al Robust high-throughput prokaryote de novo assembly and improvement pipeline for Illumina data. Microb Genom 2016;2:e000083. 10.1099/mgen.0.000083 28348874 PMC5320598

[21] Zerbino DR, Birney E Velvet: algorithms for de novo short read assembly using de Bruijn graphs. Genome Res 2008;18:821–9. 10.1101/gr.074492.107 18349386 PMC2336801

[22] Casali N, Nikolayevskyy V, Balabanova Y, et al Microevolution of extensively drug-resistant tuberculosis in Russia. Genome Res 2012;22:735–45. 10.1101/gr.128678.111 22294518 PMC3317155

[23] Li H, Handsaker B, Wysoker A, et al The sequence Alignment/Map format and SAMtools. Bioinformatics 2009;25:2078–9. 10.1093/bioinformatics/btp352 19505943 PMC2723002

[24] Harris SR, Feil EJ, Holden MTG, et al Evolution of MRSA during Hospital transmission and intercontinental spread. Science 2010;327:469–74. 10.1126/science.1182395 20093474 PMC2821690

[25] Brown AC, Bryant JM, Einer-Jensen K, et al Rapid whole-genome sequencing of Mycobacterium tuberculosis isolates directly from clinical samples. J Clin Microbiol 2015;53:2230–7. 10.1128/JCM.00486-15 25972414 PMC4473240

[26] Schliep KP phangorn: phylogenetic analysis in R. Bioinformatics 2011;27:592–3. 10.1093/bioinformatics/btq706 21169378 PMC3035803

[27] Coll F, McNerney R, Guerra-Assunção JA, et al A robust SNP barcode for typing Mycobacterium tuberculosis complex strains. Nat Commun 2014;5:4812. 10.1038/ncomms5812 25176035 PMC4166679

[28] Jombart T, Devillard S, Balloux F Discriminant analysis of principal components: a new method for the analysis of genetically structured populations. BMC Genet 2010;11:94. 10.1186/1471-2156-11-94 20950446 PMC2973851

[29] Salvatore PP, Becerra MC, Abel zur Wiesch P, et al Fitness costs of drug resistance mutations in multidrug-resistant Mycobacterium tuberculosis: a Household-Based case-control study. J Infect Dis 2016;213:149–55. 10.1093/infdis/jiv347 26092854 PMC4676541

[30] Klopper M Molecular characterization of the drug resistant tuberculosis epidemic in the Eastern Cape, South Africa, 2015 Available: https://scholar.sun.ac.za

[31] Stanley SA, Kawate T, Iwase N, et al Diarylcoumarins inhibit mycolic acid biosynthesis and kill Mycobacterium tuberculosis by targeting FadD32. Proc Natl Acad Sci U S A 2013;110:11565–70. 10.1073/pnas.1302114110 23798446 PMC3710825

[32] Eldholm V, Rieux A, Monteserin J, et al Impact of HIV co-infection on the evolution and transmission of multidrug-resistant tuberculosis. Elife 2016;5. 10.7554/eLife.16644. [Epub ahead of print: 09 Aug 2016]. PMC4978521 27502557

[33] Bogale S, Diro E, Shiferaw AM, et al Factors associated with the length of delay with tuberculosis diagnosis and treatment among adult tuberculosis patients attending at public health facilities in Gondar town, northwest, Ethiopia. BMC Infect Dis 2017;17:145. 10.1186/s12879-017-2240-0 28193183 PMC5307798

