# Supplementary figures and images for: Association between bacterial homoplastic variants and radiological pathology in tuberculosis
Source: Thorax. 2020 Jun 15;75(7):584–91. doi: 10.1136/thoraxjnl-2019-213281 (PMC7361023; doi:10.1136/thoraxjnl-2019-213281)

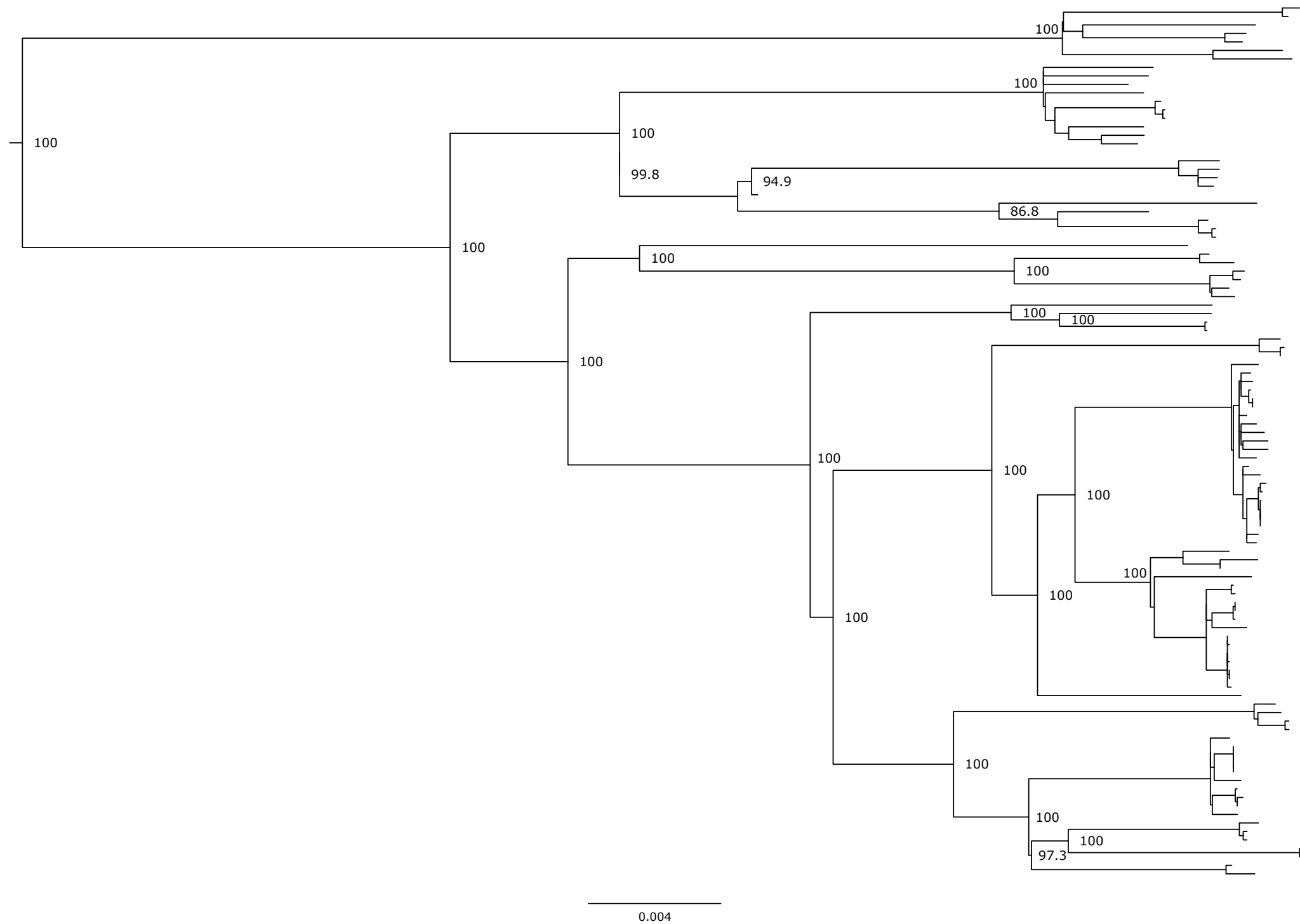

Supplement: Supplementary data [file thoraxjnl-2019-213281supp001.pdf]

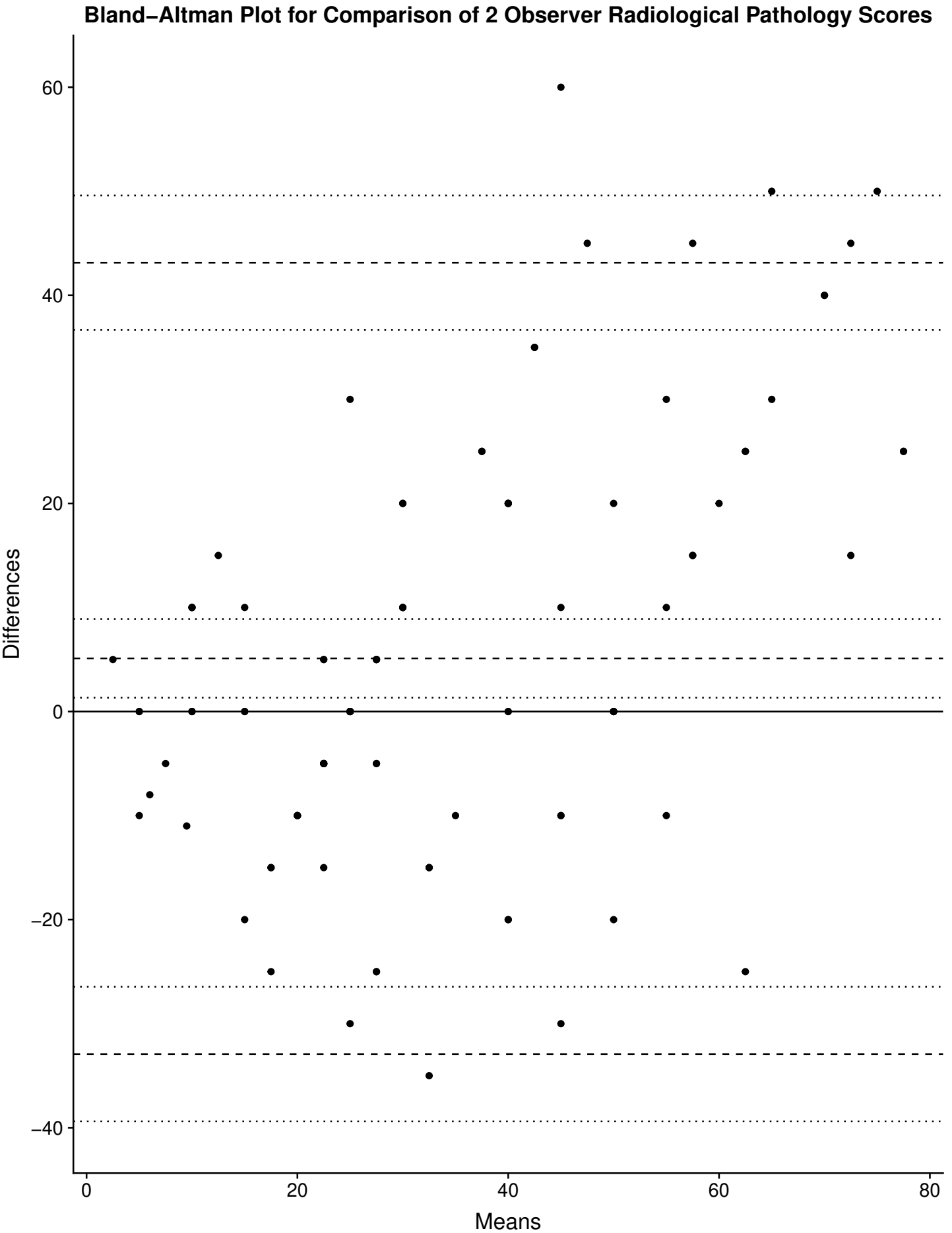

Supplement: Supplementary data [file thoraxjnl-2019-213281supp002.pdf]
